# The role of molecular diagnosis in anaphylactic patients with dual or triple-sensitization to Hymenoptera venoms

**DOI:** 10.1186/s13223-024-00885-9

**Published:** 2024-03-23

**Authors:** Mohammad Hassan Bemanian, Raheleh Shokouhi Shoormasti, Saba Arshi, Mahsa Jafari, Sima Shokri, Morteza Fallahpour, Mohammad Nabavi, Fatemeh Zaremehrjardi

**Affiliations:** 1 Department of Allergy and Clinical Immunology, Rasool-E-Akram Hospital, Iran University of Medical Sciences, Tehran, Iran; 2 Immunology, Asthma and Allergy Research Institute, Tehran University of Medical Sciences, Tehran, Iran; 3 Children’s Medical Center, Pediatrics Center of Excellence, Tehran University of Medical Sciences, Tehran, Iran; 4 Clinical Research Development Center, Imam Reza Hospital, Kermanshah University of Medical Sciences, Kermanshah, Iran

**Keywords:** Bee sting, Hymenoptera, Sensitization, IgE, Molecular diagnosis, Honey bee, Yellow jacket, Paper wasp, Bee venom allergy

## Abstract

**Background:**

The poly-sensitization to Hymenoptera venom makes it difficult to select genuine allergens for immunotherapy and increases patients’ costs. The objective of this study was to determine the culprit allergen in dual or triple-sensitized patients to three Hymenoptera venoms through molecular diagnosis and evaluating the results of incorporating the molecular diagnosis with skin tests.

**Methods:**

Thirty-two patients with anaphylactic reactions and dual or triple-sensitization to Hymenoptera venoms in skin tests entered this study. IgE-sensitization to whole extracts and molecules of *Apis mellifera* (Api m), *Vespula vulgaris* (Ves v), and *Polistes dominulus* (Pol d) was evaluated utilizing ALEX or ImmunoCAP.

**Results:**

Twenty-nine patients (90.6%) were male. IgE-sensitization to at least one of the allergenic molecules related to *Apis mellifera*, *Vespula vulgaris*, and *Polistes dominulus* was seen in 59.4, 53.1, and 21.9%, respectively. Among 32 patients, 14 (43.8) and 8 (25%), were mono-sensitized to Api m and Ves v components in ALEX, respectively. Double sensitization to Hymenoptera was identified in 18.8% of patients in ALEX. Api m 1+/Api m 2−/Api m 10− and Ves v 1+/Ves v 5+ demonstrated the most prevalent sensitizations patterns in our patients.

**Conclusions:**

The molecular diagnosis of IgE-sensitization to Hymenoptera venoms can be valuable, especially in patients who show dual or triple-sensitization in skin tests, as the ALEX results revealed mono and double-sensitization to Hymenoptera venoms in 22 and 6 patients, respectively. Regarding the high cost and adverse reactions of venom immunotherapy, especially for two or three venoms, incorporating the molecular diagnosis alongside skin tests for accurate diagnosis of the culprit venom could help decrease costs for patients.

## Introduction

Bees, wasps, sawflies, and ants belong to one of the most significant orders of insects called Hymenoptera, which includes more than 153,000 known species [1]. *Apis mellifera* (Honey Bee), *Vespula spp.* (Yellow Jacket), and *Polistes spp.* (Paper Wasp) are considered the common allergenic species of the Apidae and Vespidae families in the Hymenoptera order [2–4].

Severe immediate hypersensitivity reactions to Hymenoptera sting are recognized as a serious concern worldwide [5]. A prevalence of 0.3 to 7.5% has been reported in adults with systemic allergic reactions to Hymenoptera sting [6].

A comprehensive clinical history and a positive specific IgE to Hymenoptera allergens using in vivo [skin prick test (SPT) or intradermal] or in vitro tests and consequently the exact determination of the offending insect constitute cornerstones for the diagnosis of Hymenoptera venom allergy [4, 6]. Conventional extract-based diagnostics often show poly-sensitization to two or more venom, making the selection of genuine allergens difficult for allergen-specific immunotherapy [2, 7]. This poly-sensitization could be developed due to IgE binding to allergenic molecules with similar structures or cross-reactive carbohydrates (CCD) [2]. The production of recombinant molecules without carbohydrate moieties, molecular diagnosis of allergy, and advances in the methods for CCD inhibition led to higher diagnostic sensitivity [8, 9].

According to the WHO/IUIS Allergen Nomenclature, 12, 5, and 5 allergenic molecules were recognized for *Apis mellifera (Api m), Vespula vulgaris (Ves v), and Polistes dominulus (Pol d),* respectively [10]. A novel technique called Allergy Explorer, ALEX (Macroarray Diagnostics, Vienna, Austria) has been developed to determine the specific IgE to 282 whole extracts and allergenic molecules. The application of a CCD inhibitor in the diluent solution of this method and using recombinant molecules decrease false-positive results, especially for allergenic molecules of Hymenoptera venoms [9]. Approximately 30% of patients show dual-sensitization to Honey Bee and Wasp while demonstrating a clinical allergic reaction to one insect [11]. This study intended to determine the culprit allergen in dual and triple-sensitized patients to Hymenoptera venom, including *Apis mellifera* (Honey Bee; HB*), Vespula sp.* (Yellow Jacket, YJ*) and Polistes sp.* (Wasp Venom or Paper wasp; PW*),* through the molecular diagnosis of allergy. Another objective of the present study was to evaluate the results of incorporating the molecular diagnosis (ALEX and ImmunoCAP) with SPT and intradermal tests.

## Methods

### Patients

This research was conducted in a cross-sectional design. Thirty-two patients with anaphylactic reactions to Hymenoptera venoms referred to Rasool-e-Akram Hospital, entered the study between the years 2018 and 2020. The Ethics Committee of the Iran University of Medical Sciences approved this study (No.: IR.IUMS.FMD.REC.1398.321). Written informed consent was taken from all participants.

### Inclusion criteria

The study included all patients with anaphylactic reactions to Hymenoptera stings who had completed a refractory period of at least 4 weeks since the last anaphylactic attack. The anaphylaxis diagnosis was based on World Allergy Organization (WAO) criteria [12]. Moreover, patients should discontinue antihistamines and interfering drugs within one week before the beginning of the study.

### Exclusion criteria

This study excluded subjects with non-systemic reactions to Hymenoptera stings, mono-sensitized patients to Hymenoptera venom using skin tests, and patients with anaphylactic reactions to fire ant. Additionally, lack of consent by the patient was an exclusion criterion.

### Clinical investigations

For all patients, a questionnaire with demographic and clinical questions was fulfilled. Following the presentation of a picture of the Hymenoptera along with the geographical location, the type of Hymenopterawas recorded according to the patient's history and the pictures of the insects. According to the Mueller classification, symptoms severity is categorized into four grades [13].

### Skin prick test and intradermal test

Skin prick and intradermal tests were performed for Honey Bee Venom (*Apis mellifera*), Yellow Jacket Venom Protein (*Vespula sp*.), and Wasp Venom Protein (*Polistes sp.*) using commercial standard extracts (Jubilant Hollister Stier, Spokane, WA, USA). SPT was performed in the forearm area using a 1 µg/mL dilution of whole extracts of Hymenoptera venom [14–16].

An intradermal test was done in the forearm area using an insulin syringe with 1 µg/mL dilution of an extract. The positive and negative controls were histamine and normal saline, respectively. A 3-mm wheal greater than the negative control was considered positive [14].

### Molecular diagnosis

A three-milliliter blood sample was taken from all patients. The serum was then separated and assessed with ALEX kits (Allergy Explorer-ALEX^®^, MacroArray DX, Vienna, Austria). The ALEX is a multiplex assay for assessing total IgE and specific IgE for 282 whole extracts and allergenic molecules at the same time. One-hundred fifty-seven whole extracts and 125 molecules were coated as spots on the nitrocellulose membrane. A 400-µL diluent was added to the membrane, along with 100 µL of the serum sample. There is an interesting point regarding the presence of a CCD inhibitor in the sample diluent. Following the two-hour incubation and 3-step washings, anti-human IgE labelled with alkaline phosphatase was added. After 30-min incubation and 5-step washings, the substrate was added. The reaction was stopped by a stop solution after 8 min. Following drying the membranes, the intensity of the reaction was evaluated by Image-Explorer and Raptor software. A cutoff of > 0.3 kUA/L was considered positive. Further, if a concentration exceeded 15 kUA/L, it was considered very high [9]. IgE-sensitization to whole extracts of Api m, Pol d, and Ves v, as well as the specific IgE to allergenic molecules (Api m 1, Api m 2, Api m 10, Pol d 5 and Ves v 5) were evaluated. Moreover, the specific IgE to Ves v 1 was measured using the ImmunoCAP system (Phadia/Thermo Fisher, Uppsala, Sweden).

### Statistical analysis

Data was analyzed using statistical software, including IBM SPSS version 20 (IBM Corporation, NY, USA) and Graphpad Prism 8 (GraphPad Software, San Diego, CA, USA). Moreover, the Venn diagram was drawn by jvenn [17]. The central tendency (mean or median) and the dispersion of the data (SD or quartiles) have been determined based on the distribution of the data. Categorical variables were presented as frequency and percentage. The association between two categorical variables was evaluated by Chi square test. The correlation was determined by spearman's correlation test. Independent t-test or Mann–Whitney test were used to compare the difference of a quantitative variable between two independent groups. The agreement between some variables was determined by the kappa coefficient.

## Results

Twenty-nine (90.6%) of the 32 patients were male. The study participants had a mean (±SD) age 36.65 ± 15.15 years (min 7 and max 67 years). Twelve patients reported a history of atopic diseases (37.5%); the most common was allergic rhinitis, with a prevalence of 31.3%. A family history of atopic diseases was reported in four patients (12.5%). The mean ±SD first and last ages of anaphylactic reaction were 29.26 ± 14.69 and 34.08 ± 14.65 years old, respectively. The median (P25–P75) of total IgE was 127.5 (36–783). HB (n = 17) and YJ (n = 13) were the most common culprit insects based on the patients’ history. A total of 53% of patients with anaphylactic reactions reported two or fewer reactions, and 18.8 % reported more than five. The mean time between insect stings and the onset of symptoms was 9.63 min, while the minimum and maximum times were 1 and 30 min, respectively. Moreover, 18.8% of patients reported a history of allergic reactions to Hymenoptera stings in their family. The head, neck (31.3%), upper limb (28.1%), and lower limb (18.8) were the most frequent sting locations. Patients reported significant complaints related to their skin (90.6%), respiratory (84.4%), and cardiovascular (71.9%) systems.

Table 1 presents the patterns of allergic sensitization to the whole extract and molecules of Api m, Ves v, and Pol d using ALEX test. As the results illustrated, Api m 1 (n = 16), Ves v 1 (n = 13), Ves v 5 (n = 13), and Api m 10 (n = 11) were major allergens in this study. Table 1 demonstrated two patterns including, Ves v 1+/Ves v 5+ and Api m 1+/Api m 2−/Api m 10− patterns as common sensitization patterns in the current study. Despite a low frequency of IgE-sensitization to *Vespula vulgaris* extract in ALEX, specific IgE assays for its allergenic molecules detected patients with allergy to YJ. Table 2 presents the agreement between the history, the history after showing the Hymenoptera image, the reactivity to SPT and intradermal tests, and the results of molecular diagnosis. By history and pictures, HB was the most commonly recognized Hymenoptera. Only three patients showed positive reactivity to the SPT (YJ = 1, PW = 2), while 32 participants had a positive intradermal test. As the findings of allergic sensitization to Hymenoptera extracts demonstrated, among 32 patients with dual or triple-sensitization in the intradermal test, 15 (46.9%), 1 (3.1%), and 2 (6.25%) patients showed exclusive sensitization to whole extracts of Api m, Ves v, and Pol d in ALEX, respectively. Furthermore, 4 (12.5%) patients revealed double-sensitization to whole extracts of Hymenoptera in ALEX. Following the molecular diagnosis with the ALEX test, 14 (43.8%) and 8 (25%) patients exclusively showed IgE-sensitization to Api m and Ves v components, and 9 subjects (28.1%) demonstrated dual or triple sensitization to allergenic components of two or three venoms. A patient with negative specific IgE to all extracts and molecules has also been observed. Finally, a total of 22 patients (68.8%) exhibited IgE specific to at least one whole allergen extract using the ALEX test, while it identified 31 (97%) patients with allergenic molecules of Hymenoptera. A kappa coefficient of 0.68, 0.48, and 0.07 was obtained for IgE sensitization to Api m, Ves v, and Pol d based on ALEX and the self-reported history of patients. Intradermal and ALEX methods showed the highest agreement for HB. Among the studied Hymenoptera, it seems that the PW, with 76.7%, shows the highest false-positive sensitization in the intradermal test.

**Table 1:** The frequency and percentage of different patterns of IgE-sensitization to whole extract and molecules of *Apis mellifera*, *Vespula vulgaris* and *Polistes dominulus* in ALEX test

IgE sensitization to whole extracts of hymenoptera in ALEX test									
IgE sensitization pattern	Total	Single-Sensitization to Api m extract (n = 15)	Single-sensitization to Ves v extract (n = 1)	Single-sensitization to Pol d extract (n = 2)	Double-sensitization to Api m and Ves v extracts (n = 0)	Double-sensitization to Api m and Pol d (n = 2) extracts	Double-sensitization to Ves v and Pol d extracts (n = 2)	Multi-sensitization to Api m, Ves v and Pol d Extracts (n = 0)	All extracts negative (n = 10)
IgE sensitization patterns to allergenic molecules of Hymenoptera in ALEX test and ImmunoCAP									
Api m 1+/Api m 2−/Api m 10−	8 (25)	5 (33.3)	0	0	0	1 (50)	1 (50)	0	1 (10)
Api m 1−/Api m 2+/Api m 10−	0	0	0	0	0	0	0	0	0
Api m 1−/Api m 2−/Api m 10+	3 (9.4)	3 (20%)	0	0	0	0	0	0	0
Api m 1+/Api m 2+/Api m 10−	0	0	0	0	0	0	0	0	0
Api m 1+/Api m 2−/Api m 10+	5 (15.6)	4 (26.7)	0	0	0	1 (50)	0	0	0
Api m 1−/Api m 2 + /Api m 10 +	0	0	0	0	0	0	0	0	0
Api m 1+/Api m 2+/Api m 10+	3 (9.4)	3 (20%)	0	0	0	0	0	0	0
Api m 1−/Api m 2−/Api m 10−	13 (40.6)	0	1 (100)	2 (100)	0	0	1 (50)	0	
Ves v 1+/Ves v 5−	4 (12.5)	1 (6.7)	0	1 (50)	0	0	0	0	2 (20)
Ves v 1−/Ves v 5+	2 (6.7)	0	0^a^	0	0	1 (50)	0	0	1 (10)
Ves v 1+/Ves v 5+	9 (30)	1 (6.7)	0^a^	1 (50)	0	1 (50)	2 (100)	0	4 (44.4)^a^
Ves v 1−/Ves v 5−	15 (46.9)	13 (86.7)	0	0	0	0	0	0	2 (20)
Pol d 5+	7 (21.9)	0	0	2 (100)	0	2 (100)	2 (100)	0	1 (10)
Pol d 5−	25 (78.1)	15 (100)	1 (100)	0	0	0	0	0	9 (90)

^a^The specific IgE to Ves v 1 was unavailable for two patients. Moreover, the specific IgE to Ves v 5 was positive for both patients. Therefore, the pattern of IgE sensitization for these two patients could be Ves v 1+/Ves v 5+ or Ves v 1−/Ves v 5+

**Table 2:** The agreement of patient history, picture of Hymenoptera, and the SPT, intradermal and ALEX results

	History N (%)	Picture N (%)	SPT (extract) N (%)	Intradermal (extract) N (%)	ALEX (extract) N (%)	ALEX and ImmunoCAP (molecule) N (%)
Honey bee venom (*Apis mellifera)*	13 (40.6)	10 (31.3)	0 (0)	0 (0)	15 (46.9)	14 (43.8)
Yellow jacket venom *(Vespula vulgaris)*	8 (25)	5 (15.6)	1 (3.1)	0 (0)	1 (3.1)	8 (25)
Paper wasp (*Polistes dominulus)*	1 (3.1)	1 (3.1)	2 (6.3)	0 (0)	2 (6.3)	0 (0)
Honey bee and yellow jacket	2 (6.3)	0 (0)	0 (0)	2 (6.3)	0 (0)	2 (6.3)
Yellow jacket and paper wasp	1 (3.1)	1 (3.1)	0 (0)	1 (3.1)	2 (6.3)	4 (12.5)
Honey bee and paper wasp	0 (0)	0 (0)	0 (0)	0 (0)	2 (6.3)	0 (0)
Honey bee and yellow jacket and paper wasp	2 (6.3)	2 (6.3)	0 (0)	29 (90.6)	0 (0)	3 (9.4)

**Fig. 1:**
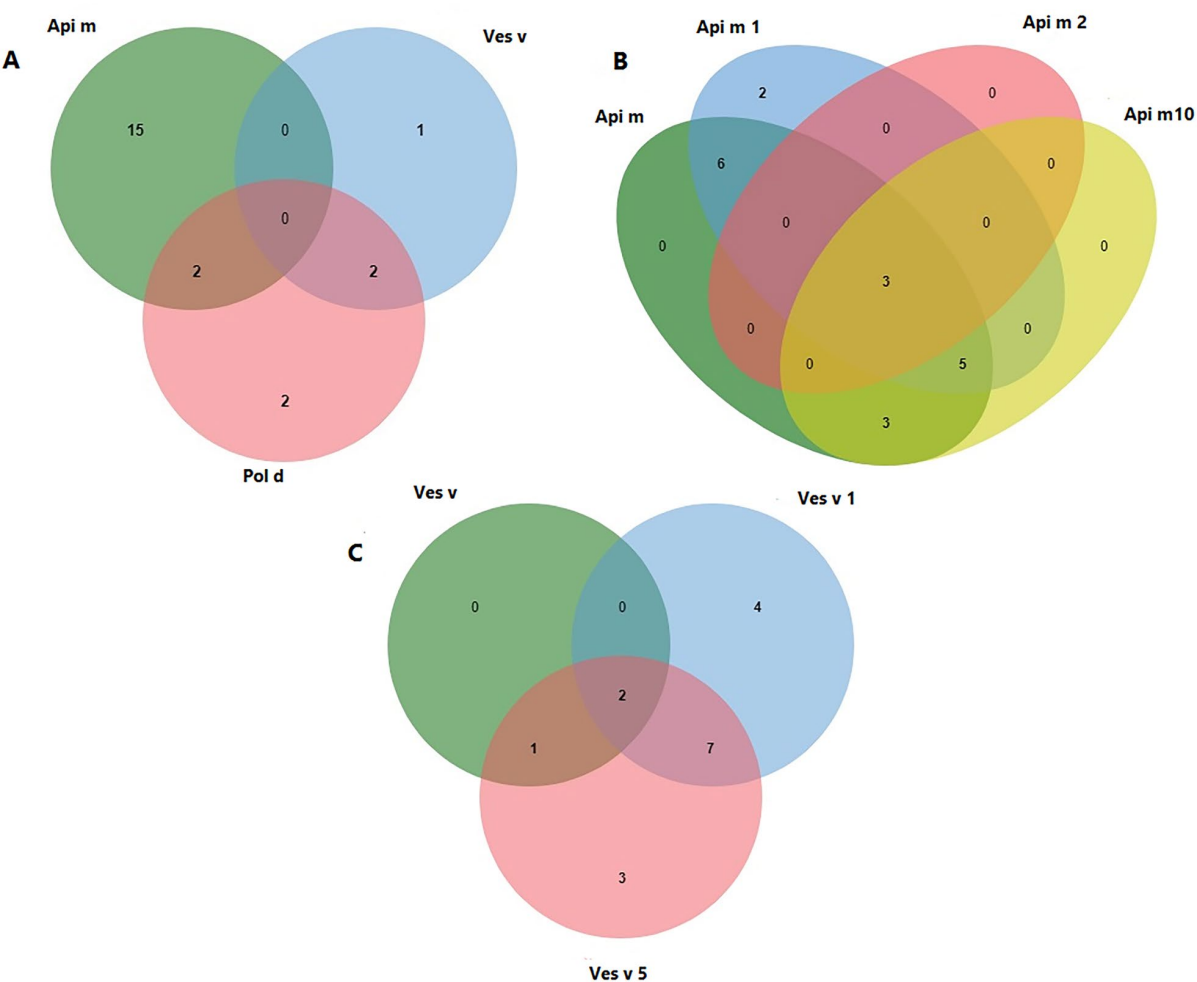
Venn diagram for the IgE-sensitization to whole extracts of *Apis mellifera*, *Vespula vulgaris* and *Polistes dominulus* in ALEX (**A**); Venn diagram for the IgE-sensitization to allergenic molecules of *Apis mellifera in ALEX* (**B**); Venn diagram for the IgE-sensitization to allergenic molecules of *Vespula vulgaris* with ALEX and ImmunoCAP (**C**). jvenn was used to draw Venn diagrams [17]

Figure 1A–C demonstrated the IgE-sensitization to whole extracts of Api m, Ves v, and Pol d; IgE-sensitization to allergenic molecules of Api m as well as IgE-sensitization to allergenic molecules of Ves v, respectively. Contrary to the intradermal results, none of the patients showed IgE sensitization to all three Hymenoptera whole extracts, and exclusive IgE-sensitization to Api m extract was considered the most prevalent (n = 15) (Fig. 1A). Three patients simultaneously showed allergic sensitization to Api m extract and it’s three molecules, as shown in Fig. 1B. None of the subjects showed mono-sensitization to Api m 2. There were four and two individuals with Ves v 1+/Ves v 5− and Ves v 1−/Ves v 5+ sensitization patterns, respectively (Table 1). It should be noted that the results of specific IgE to Ves v 1 were unavailable for two patients. Among 17 patients with sensitization to Ves v, only 3 patients were identified with the whole extract of Ves v, while 14 patients with allergy to Ves v were identified using allergenic molecules of Ves v.

**Fig. 2:**
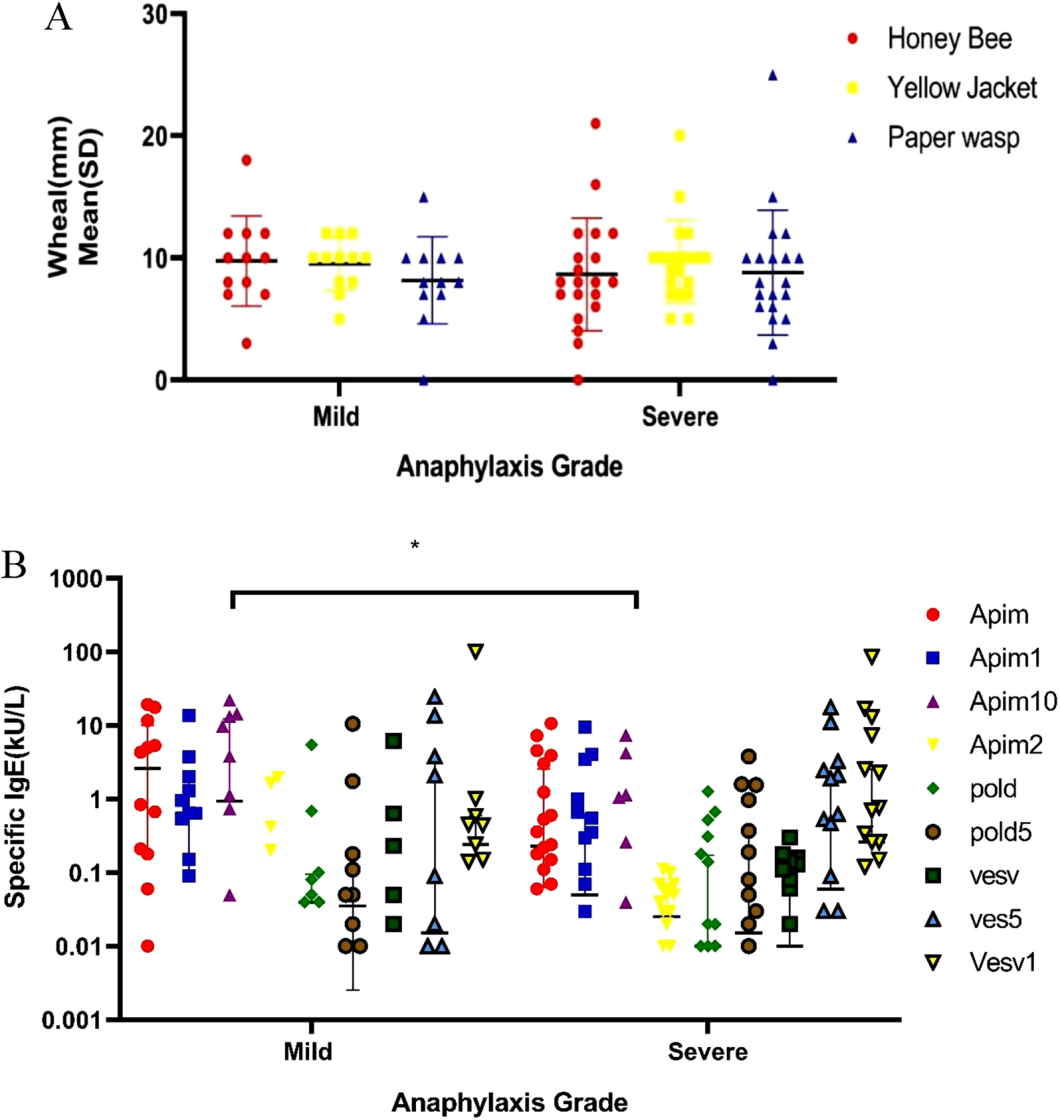
**A** Wheal diameter of intradermal test for Hymenoptera venoms according to anaphylaxis grades. **B** The specific IgE concentration to allergenic extracts and molecules of Hymenoptera venoms according to anaphylaxis grades

**Fig. 3:**
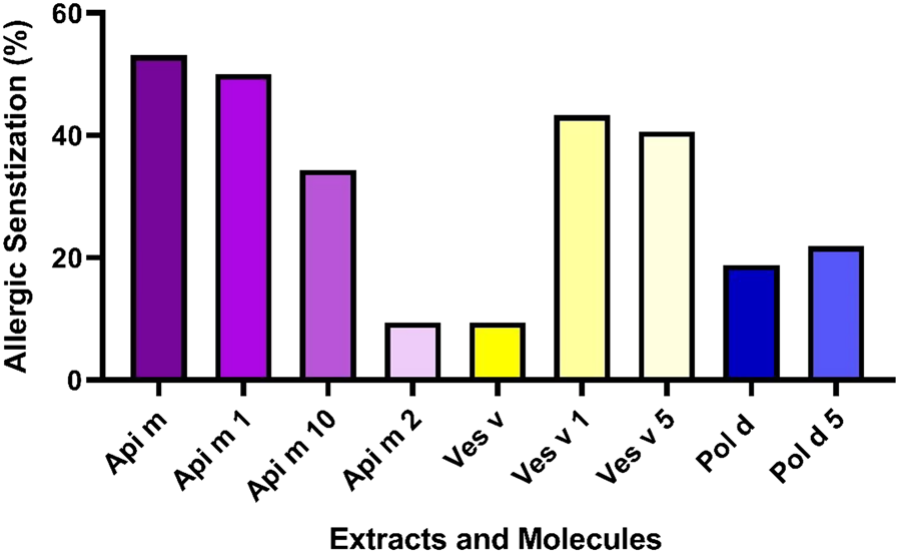
IgE-sensitization to allergenic extracts and molecules of different Hymenoptera venoms

Figure 2 displays the wheal diameter of the intradermal test for Hymenoptera venoms and the specific IgE concentration to allergenic extracts and molecules of Hymenoptera venoms according to anaphylaxis grades. No significant difference was found between the wheal diameter of three Hymenoptera and the grade of anaphylaxis. Additionally, patients with mild anaphylaxis had a higher specific IgE to Api m 10 than those with severe anaphylaxis (P = 0.02). Figure 3 shows IgE-sensitization to all studied allergenic molecules in this study.

Table 3 demonstrates the relationship between the time interval since the last reaction and the frequency of IgE-sensitization to extracts and molecules of Hymenoptera. With increasing time, the IgE reactivity for most allergens decreased, although it was only significant for Api m1. Moreover, this decline has not been shown for Ves v 1. Specific IgE to Api m whole extract significantly was correlated with specific IgE to Api m 1, Api m 2, and Api m 10 (P < 0.001), although the highest correlation coefficient was related to Api m 1 (r = 0.718) and Api m 10 (r = 0.686). Moreover, specific IgE to Ves v whole extract showed a meaningful correlation with Ves v 5 (P = 0.005, r = 0.475) not Ves v 1(P = 0.14). A significant correlation was found between sIgE to Pol d whole extract and Pol d 5 (P < 0.001, r = 0.78).

**Table 3:** Relationship between time interval from last reaction with frequency of IgE-sensitization to extracts and molecules of Hymenoptera

Anaphylaxis	Api m	Api m 1	Api m 2	Api m 10	Ves v	Ves v 1	Ves v 5	Pol d	Pol d5
< 1 y N = 17	10 (58.8)	12 (70.6)	1 (5.9)	5 (29.4)	3 (17.6)	6 (37.5)	6 (35.3)	5 (29.4)	5 (29.4)
1–4 y N = 9	5 (55.6)	3 (33.3)	2 (22.2)	5 (55.6)	0 (0)	2 (25)	4 (44.4)	0 (0)	1 (11.1)
> 4 y N = 6	2 (33.3)	1 (16.7)	0 (0)	1 (16.7)	0 (0)	5 (83.3)	3 (50)	1 (16.7)	1 (16.7)
P value	0.57	0.04	0.26	0.32	0.40	0.09	0.80	0.21	0.63

## Discussion

The current study found that the molecular diagnosis of IgE-sensitization to Hymenoptera venoms is valuable for the detection of culprit venom, especially in patients with dual or triple-sensitization. As the ALEX results demonstrated, mono- and double-sensitization to Hymenoptera venoms were observed in 22 and 6 patients, respectively. The most common Hymenoptera causing anaphylaxis were HB and YJ. Ves v 1 + /Ves v 5 + and Api m 1 + /Api m 2−/Api m 10− patterns were also identified as major sensitization patterns in these patients.

In our study, as well as the studies of Hirata et al., Seob Shin et al., and Bemanian et al., males were more frequent than females [18–20]. Gelincik et al. found that males and females had the same prevalence of Hymenoptera venom allergies in Istanbul [21]. This difference may be attributed to the culture effect such as the influence of wearing a hijab on women in some countries, and the higher frequency of men in difficult jobs such as agriculture, animal husbandry, and beekeeping. According to the Blank et al. study, being male could be considered a predictor of having positive IgE specific to HB [22]. Another study suggested that being male and older were risk factors for developing severe allergic reactions to Hymenoptera [23].

As the findings demonstrated, twelve patients (37.5%) reported a history of atopic diseases, most of which were allergic rhinitis (31.3%). This prevalence of allergic rhinitis is a little higher than in the general population (28.3%) [24]. In a study by Yavuz et al., bronchial asthma and allergic rhinitis were identified in 25% and 17.1% of children with Hymenoptera venom allergies, respectively [25]. As mentioned in the literature, atopic diseases could not be considered a predictor of developing hypersensitivity to Hymenoptera sting [21, 26]. Compared with individuals with mono-sensitization to Hymenoptera venom, an insignificantly higher prevalence of atopic diseases (28.8% vs. 42.1%) was observed in patients with dual-sensitization [27]. Although our study found a lower prevalence of atopic diseases in patients with dual or triple sensitization to Hymenoptera venom.

Ten (31.3%) patients reported a bite on the head and neck. The bite mainly affected the neck and upper limbs, although other limbs were involved as well. A study by Bemanian et al. reported that bites most frequently occurred in the upper limbs, followed by the head and neck [28]. The findings of the current study are consistent with those of previous studies regarding the major clinical manifestations [19, 25].

The lower sensitivity of SPT for Hymenoptera venoms leads to the use of the intradermal test to identify offending venom [29]. Based on our inclusion criteria and the intradermal test results, double and triple-sensitizated patients were included. Consequently, it is difficult to determine the genuine insect for allergen-specific immunotherapy. Using recombinant species-specific major molecules in Hymenoptera venoms and CCD inhibition may improve the ability to distinguish dual or triple sensitization from cross-reactivity [27].

In our study, 19 patients were identified with IgE sensitization to *Apis mellifera* components, of which 42.1% showed IgE-sensitization to more than one component, 57.9% to only one component, and 15.8% to all three components. In Kohler et al.’s study, IgE-sensitization to more than one molecule was 74.3%, while 9.7% showed positive specific IgE to all the studied allergenic molecules of *Apis mellifera* [30]. A 59.4% prevalence of IgE sensitization to at least one molecule of HB was found in the present study, while Kohler reported an 89.6% prevalence [30]. This difference could result from the difference in sample size and the number of allergenic molecules investigated between the two studies. As Bilo et al. stated, the evaluation of more allergic molecules increases the detection of the offending Hymenoptera [31]. According to our study, the most common sensitization pattern in HB-allergic patients was exclusive sensitization to Api m1 (Api m 1+/Api m 2−/Api m 10−) (25%). The next patterns were Api m 1+/Api m 2−/Api m 10+, Api m 1−/Api m 2−/Api m 10+ and Api m 1+/Api m 2+/Api m 10 + . In Kohler et al.’s study, these patterns were 11.81%, 3.47%, 4.17%, and 1.39%, respectively, although they also studied other molecules, including Api m 3, Api m 4 and Api m 5. Api m1+/Api m 2+/Api m 3+/Api m 4−/Api m 5+/Api m 10+ and Api m 1+ exclusive pattern was more common in the Kohler study [30].

Among 32 patients with positive intradermal reactions to Yellow Jacket Venom Protein extract, three showed positive specific IgE to whole extract from *Vespula vulgaris* in the ALEX test, so the intradermal test and whole extract of YJ in the ALEX test have poor agreement in this study. It could be due to the low amount of Ves v 1 and Ves v 5 molecules in the whole extract of YJ in the ALEX. Eikan et al. obtained an agreement of 79% between the skin tests and specific IgE assay [32]. The sensitivity of the specific IgE assay to the whole extract of YJ (*Vespula spp*.) was 83% using ImmunoCAP [33]. The differences between our study and other studies may be due to differences in measurement tools or the small sample size. On the other hand, different time intervals between the bite and the specific IgE assay can affect the results [34]. An evaluation of the allergenic components of *Vespula vulgaris* enabled us to identify seventeen patients while three of them were only positive in the whole extract in ALEX.

In the study of Strum et al., the molecular diagnosis was performed on 26 patients with allergy to YJ that showed no positive specific IgE to YJ whole extract. It's interesting that their study demonstrated positive specific IgE to rVes v 5 in 17 subjects (65.4%) [35]. In another study by Gawik et al., among 8 patients with negative sIgE to the whole extract of Vespula, 3 and 2 showed IgE sensitization to rVes v 5 and rVes v 1, respectively [36]. In our study, seven out of 29 patients with negative sIgE to the whole extract of *Vespula vulgaris (Ves v)* in ALEX had dual-sensitization to Ves v 5 and Ves v 1. Additionally, nine patients with a positive intradermal test for YJV showed a Ves v 5+/Ves v 1+ pattern. These variations may be explained by methodological or geographic differences as well as low levels of some molecules in the whole extract of YJ [37]. According to the Vos et al. study, ImmunoCAP showed positive sIgE to YJV whole extract in 83.4% of patients with allergy to YJ. Moreover, IgE sensitization to Ves v1 and Ves v 5 was identified in 44.2% and 89.9% of patients, respectively. Overall, 96.1% of patients were identified as the result of sIgE assays for Ves v 1 and Vesv5 [33]. In the same way, the present study identified 17 patients with sIgE to Ves v, Ves v 1 and Ves v 5.

In our study, the prevalence of Pol d 5 sensitization was 21.9% while Bilo et al. reported a prevalence of 69–72% [31]. This difference could be due to the geographic distribution of Hymenoptera and, of course, the sample size in our study. Furthermore, major allergen of Iranian patients may be different with other populations. In some studies, subjects with a positive skin test or sIgE to the whole extract were considered the baseline group. By contrast, our study included only patients who had positive skin tests. In Shin et al.’s study, there was a positive correlation between sIgE to the extract and the allergenic molecule of Pol d (r = 0.757) [19].

Molecular profiles and patient reports of Hymenoptera stings revealed that 15 patients had a history of stings along with IgE positive to allergenic molecules of the same reported insect. Despite a moderate agreement between the results of a specific IgE assay and the patients’ ability to identify offending insects [38], it could not alone help identify the causative insect. In the Reisman et al. study, among 46 patients with positive sIgE to bees, 26 individuals recognized the culprit Hymenoptera [38].

Although Api m 1 and Api m 2 seem to be associated with less severe anaphylactic reactions, there was no association between IgE sensitization to bee extracts and molecules and anaphylactic reaction severity. Gawlik et al. explored a positive correlation between specific IgE to Ves v 1, specific IgE to the whole extract of YJ and HB, and Api m1 with the severity of anaphylaxis [36].

As the findings of allergic sensitization to HB, YJ and PW extracts demonstrated, among 32 patients with dual or triple-sensitization in the intradermal test, 15 (46.9%), 1 (3.12%) and 2 (6.3%) patients showed exclusive sensitization to the whole extracts of Api m, Ves v, or Pol d, respectively, while 4 (12.6%) patients revealed positive specific IgE to two Hymenoptera. Following the molecular diagnosis with the ALEX test, 14 (43.8%) and 8 (25%) patients exclusively showed positive sensitization to Api m and Ves v components, and 9 subjects (28.1%) demonstrated allergic sensitization to allergenic components of two or three types of Hymenoptera (Table 2). In the study of Selb et al. 98 and 69 (70.4%) out of 255 patients showed dual-sensitization to allergen extract and allergenic molecules, respectively [39]. In our study, nine patients (30%) remained dual or triple-sensitized according to the ALEX test using both whole extract and components. This difference could be due to the evaluation of more molecules in the Selb et al. study [39]. Although, we should take note of the fact that all participants had positive allergic sensitization to the extracts in their study.

As the current study results demonstrated, a meaningful correlation was observed between sIgE to Api m and Api m 1 and Api m 10; Ves v and Ves v 5; and Pol d, and Pol d 5. Similarly, Hirata et al., study indicated the same result between Api m and its molecules, especially Api m 1 (r = 0.98) [20].

In light of the high cost of venom immunotherapy (VIT), especially for two or three venoms [40] and systemic anaphylactic reactions [41], the molecular diagnosis could help patients in reducing these issues. Using the intradermal test alone, patients would have to undergo VIT for at least two or three Hymenoptera, while the molecular diagnosis significantly decreases the number of VITs. Using ALEX, we observed mono- and double-sensitization to Hymenoptera venom in 22 and 6 patients, respectively. Moreover, this test meaningfully improved immunotherapy specificity towards related venom. Inhibiting cross-reactive carbohydrates in ALEX and identifying cross-reactive proteins could explain the differences between the two methods.

Since all cases of *Polistes dominulus* sensitization was associated with *Vespula vulgaris* sensitization, cross-reactivity is likely. Six patients showed positive specific IgE to Ves v 5 and Pol d 5. Ves v 5 is probably the cross-reactive component. The results are consistent with a Perez study that points out their high homology [8]. By identifying and evaluating genuine molecules of *Polistes dominulus,* true sensitization could occur.

We did face two limitations in our study, which were that some molecules, such as Api m 3 and Api m 5, were not evaluated and that the sample size was small. Expanding the repertoire of allergenic molecules could improve the effectiveness of this method for diagnosing Hymenoptera hypersensitivity.

## Conclusion

These results suggest that the molecular diagnosis of IgE-sensitization to Hymenoptera venoms is useful, especially for patients with dual or triple-sensitization. As a result of component-resolved diagnostics with ALEX, CCD markers are inhibited, cross-reactive molecules are identified, and culprit venom can be more accurately diagnosed. Moreover, VIT costs are minimized for patients with Hymenoptera allergies.

## Data Availability

The datasets used and/or analysed during the current study are available from the corresponding author on reasonable request.
